# Teaching principles of translational science to a broad scientific audience using a case study approach: A pilot course from the National Center for Advancing Translational Sciences

**DOI:** 10.1017/cts.2022.374

**Published:** 2022-03-21

**Authors:** Jessica M. Faupel-Badger, Amanda L. Vogel, Shadab F. Hussain, Christopher P. Austin, Matthew D. Hall, Elizabeth Ness, Philip Sanderson, Pramod S. Terse, Xin Xu, Krishna Balakrishnan, Samarjit Patnaik, Juan J. Marugan, Udo Rudloff, Marc Ferrer

**Affiliations:** 1 National Institutes of Health, National Center for Advancing Translational Sciences, Bethesda, MD, USA; 2 Flagship Pioneering, Cambridge, MA, USA; 3 National Institutes of Health, National Cancer Institute, Bethesda, MD, USA

**Keywords:** Education, research training, workforce, online learning, evaluation

## Abstract

There are numerous examples of translational science innovations addressing challenges in the translational process, accelerating progress along the translational spectrum, and generating solutions relevant to a wide range of human health needs. Examining these successes through an education lens can identify core principles and effective practices that lead to successful translational outcomes. The National Center for Advancing Translational Sciences (NCATS) is identifying and teaching these core principles and practices to a broad audience via online courses in translational science which teach from case studies of NCATS-led or supported research initiatives. In this paper, we share our approach to the design of these courses and offer a detailed description of our initial course, which focused on a preclinical drug discovery and development project spanning academic and government settings. Course participants were from a variety of career stages and institutions. Participants rated the course high in overall value to them and in providing a unique window into the translational science process. We share our model for course development as well as initial findings from the course evaluation with the goal of continuing to stimulate development of novel education activities teaching foundational principles in translational science to a broad audience.

## Introduction

Translational science seeks to surmount long-standing and pervasive challenges that stymie research across all diseases and conditions in order to accelerate and enhance the translational process [1]. Examples of recent translational science innovations include models that better predict efficacy, pharmacokinetics, and toxicity of new compounds in areas related to pain, addiction, and overdose, new approaches that increase clinical trial enrollment, innovations in developing and testing potential treatments for rare diseases, and novel tools and methods to rapidly generate and disseminate knowledge related to urgent public health needs [2–8]. Across these examples, the overarching goal is to generate solutions that can be applied to advance research across a broad range of research initiatives. Developing these solutions requires that translational scientists examine the research process to identify shared challenges across scientific initiatives (e.g., low enrollment in clinical trials, lack of models that accurately mirror human disease progression) and apply methods and generalizable principles from prior successful research efforts to new settings.

The examples cited above, among many others, demonstrate there is considerable accumulated experiential knowledge in the scientific community regarding how to develop and implement successful translational science innovations. However, this overarching knowledge about the translational process has not been formalized into a curriculum where it can be easily taught to broad audiences representing current and future translational scientists. The next steps for translational science education are to first formalize the generalizable core knowledge that defines and enables a translational science approach and then demonstrate how these approaches can be applied broadly to develop strategies to overcome common challenges to all research across the translational spectrum. Given the translational research community’s plethora of experiential knowledge, new education activities can be developed that build upon recent research advances by examining how a specific initiative or advancement offers insights into the translational process, common translational science challenges encountered and how these were addressed, and generalizable lessons learned or solutions developed that can be articulated, taught to others, and applied to a wide variety of research projects.

These proposed new education activities would augment existing translational science education and training efforts. Examples of current curriculum components in translational science education and training programs include an emphasis on specific skills outlined in the core competencies by the CTSA Education and Career Development key function committee and developing awareness of different aspects or phases of the translational process (e.g., regulatory aspects, dissemination, and implementation) [9–12]. These are all important activities in developing translational scientists who conduct rigorous and ethical research, are conversant across disciplines, and understand the team dynamics necessary to advance research projects. There is also a need to add education activities that focus specifically on conveying foundational knowledge in the field of translational science, including a holistic examination of the many scientific and operational facets of the translational process and enhancing understanding of the core principles and effective practices applicable to advancing all translational research across the translational spectrum, including basic, preclinical and clinical research, implementation science, and public health research [13]. Indeed, this need to identify, aggregate, and disseminate effective practices for translational science and teach these to the broader clinical and translational science workforce has also been echoed in stakeholder responses to a recent Request for Information (RFI) on enhancing the Clinical and Translational Science Awards (CTSA) Program funded by the National Center for Advancing Translational Sciences (NCATS) [14,15].

Developing this knowledge base would further distinguish the field of translational science and could also be used to expand the number of individuals in the biomedical research workforce who are exposed to translational science concepts through a wide range of education activities [16]. A significant focus of the CTSA hubs is providing rigorous biomedical research training to hundreds of early-career translational scientists each year [17–19]. Not only would these individuals benefit from additional formalized curricula teaching translational science principles, but these same curricula could be disseminated more broadly to reach scientists beyond the CTSA hubs, as well as those at different stages in their education and training (e.g., undergraduates or mid-career professionals from different disciplines).

Recognizing this need to develop new education activities that build upon recent translational science successes and are accessible to a broad audience, the NCATS Education Branch sought to develop online courses that leverage case studies of highly effective translational research initiatives to teach core concepts of translational science. The case study approach was selected because of the value of this method to bring theoretical concepts to life via real-world examples and to allow individuals from a wide range of education and career stages to reflect on the content based on their own experiences [20]. This approach is also ideal for adult learners who seek out education opportunities that allow them to draw on their experiences to date and are relevant to their immediate professional needs or interests [21].

The case study-based teaching approach has long been used across a variety of disciplines (e.g., law, business, medicine) [20]. In the context of teaching translational science, this approach is ideal for demonstrating the breadth, connectivity, and multidisciplinary nature of translational science and has already been used successfully elsewhere [22]. Here, we emphasize using the case study approach to present a holistic view of a translational science success story through conveying salient aspects of a research project and the multilevel interacting factors that influence its success (e.g., scientific goals, team composition and interactions, organizational policies, partnership dynamics). The approach draws out core concepts in translational science, including principles, approaches, and strategies contributing to success, that are exemplified in the case and are generalizable to translational science initiatives broadly.

The case study approach can be applied to teach core concepts based on any number of research success stories, creating the opportunity to explore a broad range of translational science challenges and solutions occurring at all points along the translational spectrum. To date, the NCATS Education Branch has produced three courses designed around case studies of highly successful NCATS-led or supported translational science efforts. These courses have focused on different phases of the translational science spectrum (e.g., preclinical research, clinical research, population health research) and/or specific diseases and conditions (e.g., cancer, COVID-19 pandemic, rare diseases). The overall philosophy guiding the development of these courses and key learning objectives are presented in Table 1. In this paper, we will discuss the development and implementation of an initial online course, offered in both summer and fall 2020, entitled, “MEDI 501: Principles of Preclinical Translational Science, A case study from Cancer Drug Discovery and Development,” (hereafter referred to as MEDI 501). Here, we share the design of MEDI 501 and initial findings about course participants and their satisfaction with the course in its first two sessions.

**Table 1.** Guiding philosophy for translational science courses developed by the NCATS education branch and objectives for all National Center for Advancing Translational Sciences (NCATS) education branch translational science courses

**Philosophy guiding development of NCATS education branch translational science courses**
Our courses will: • Emphasize the scientific and operational principles guiding translational science approaches. • Demonstrate how translational science principles are exemplified across a wide range of scientific initiatives and research projects. • Teach translational science in a manner that is accessible to individuals at all career stages. • Use examples of translational science innovations, including in-depth case studies and other formats to demonstrate how translational science principles applied to real-life examples. • Leverage internal expertise at NCATS to share the successful translational research activities and impart our staff members’ experiential knowledge of translational science. • Conduct rigorous course evaluations to assess the effectiveness of our courses for teaching translational science concepts. • Apply evaluation data to continually revise and improve course design, content, and delivery.
**Objectives for all NCATS education branch translational science courses**
Students will be able to: • Compare and contrast the definitions and goals of translational research and translational science. • Identify a range of scientific and operational translational science principles that can be applied to enhance translational research projects and initiatives. • Learn about the translational spectrum, and strategies needed to accelerate advancement along the spectrum, to enable scientific discoveries to lead to effective interventions in humans. Understand the importance of collaboration among different disciplines, stakeholders, and agencies – including government, universities, and industry – in advancing translational research, and strategies for how to facilitate effective cross-disciplinary, inter-agency, and team-based collaboration.

## Course Development: Case Study and Course Faculty

MEDI 501 was designed around a case study of a drug discovery and development project conducted as a collaboration among NCATS, Northwestern University, University of Kansas, and the National Cancer Institute (NCI) of the NIH. This collaboration resulted in a promising compound, metarrestin, to treat advanced metastatic cancer, which is currently in phase 1 clinical trials [23]. This internal NCATS project was selected for a case study in part because it demonstrated a remarkable range of effective practices in translational research spanning government and academic settings. In addition, the in-house experts who conducted the work were able to tell the story of the case, themselves, making for a powerful case study. Toward this end, the course focused on the components of the metarrestin project led by scientists from NCATS and NCI. In addition, these scientists were engaged from the beginning to help conceptualize and design the course.

The course began as a pilot comprising an in-person seminar and discussion series with NCATS internal fellows (postbaccalaureate, graduate student, and postdoctoral) working on-site at the NCATS research facilities in Maryland. The publication of the core preclinical research documenting the effect of metarrestin on metastasis and possible mechanisms underpinning this effect, along with preparations to initiate a phase 1 clinical trial, provided rich material from which to showcase a contemporary research project as it was unfolding and to highlight larger translational science concepts including challenges and effective approaches to mitigate them. A core group of scientists (MF, SP, RU, JM, PT, XX, and BK) served as faculty, and together with NCATS Education Branch staff (JFB), spent 5 months discussing key aspects of the project to highlight in the seminar series, and the best approaches for doing this (Supplementary Table 1). These scientists developed lectures describing the science at the heart of the case and the translational science approaches that advanced the research. Additionally, they made connections among specific strategies for success used in this project with broadly generalizable principles for effective translational research.

The seminar series was delivered over a 6-week period and included weekly lectures and assigned readings. Informal written and verbal post-course feedback indicated NCATS’ internal fellows valued the course and were interested in participating in future case study-based courses on translational science. In addition, course faculty expressed interest in continuing to share their experiential knowledge. The combined enthusiasm of NCATS’ fellows and scientists indicated that what started as a pilot course had potential for enhancing translational science education more broadly.

In order to develop an online course for a broad audience that would not have the same knowledge as NCATS’ fellows with regard to internal NCATS research programs and translational science, nor access to faculty for follow-up discussions, more lectures and faculty were added to the course. This both expanded upon and enhanced the presentations given by the original faculty and added entirely new content (Supplemental Table 1). The original faculty members – who were part of the project research team – included biologists, chemists, toxicologists, pharmacologists, and physician-scientists. For the online course, they were joined by additional NCATS (ALV, CPA, MDH, and PS) and NCI (EN) staff members who provided lectures on translational science, NCATS internal capabilities, team science, clinical trials design, and first-in-class drug development. Together, the faculty members’ expertise covered the entire spectrum of preclinical research activities necessary to bring drug candidates to phase 1 clinical trials, and a range of translational science strategies applicable to a wide variety of preclinical research projects.

Preparing to deliver an online course required developing slides aligned with the goals of the course and online format, video recording lectures, developing a course website, creating an expanded course reading list, creating student assessments, producing live Q and A sessions with faculty, and developing and obtaining IRB approval for the course evaluation (Supplementary Table 1). Ultimately, the online course offered a 7-week series of prerecorded lectures that provided a seamless story of the case study and an overarching perspective on all aspects of the project, from its origins to all aspects of the preclinical research, first in human clinical trials, teamwork, partnerships, and legal agreements. The course covered a decade of work or more, in 7 weeks.

## Online Course Implementation

The resulting one-credit course was offered online through a partnership with the Foundation for Advanced Education in the Sciences (FAES), which provides face-to-face and online classes focused on a variety of sciences and science-related areas and is located on the NIH campus [24]. FAES provided additional advertising for the course, managed registration, and provided technical assistance with recording lectures via Zoom, online course software (i.e., Panopto), and the Learning Management System (i.e., Canvas). The course registration fee was $50 and was paid directly to FAES to cover costs associated with these administrative activities. NCATS Education Branch staff (JFB and ALV) served as course instructors and responded to student inquiries, graded all assignments, monitored discussion boards, and facilitated two live sessions during the course.

MEDI 501 was designed for students at different career and training stages with varying degrees of prior knowledge relevant to translational science, preclinical drug discovery and development, and the cancer focus of the case study. As such, the course was advertised broadly through postings to professional society listservs (e.g., Association of American Medical Colleges), NIH listservs and email lists that reach both internal and external audiences (e.g., NIH Science of Team Science listserv), and the NCATS and FAES websites. The postings reached NCATS stakeholders and extramurally funded scientists and trainees, internal NIH audiences, and the broader scientific community (e.g., foundations, patient advocacy groups). Both the summer and fall 2020 sessions of this course reached capacity within days of registration opening. Registration was on a first-come, first-served basis, and all registrants were enrolled in the course for credit with expectations for completion of all required course assignments and receiving a letter grade at the end of the course. The summer session had an enrollment cap of 50 students. NCATS Education Branch set this limit for the first run of the course to manage the expected workload for instructors. The fall session enrollment cap was increased to 65 to include all individuals on the waitlist from the summer session and new registrants. For both sessions, enrollment was filled within days after course registration went live. A total of 112 students participated across the 2 sessions.

The online course design enabled students to participate asynchronously and included regular touchpoints throughout the 7 weeks. Lectures and other course materials were organized into seven modules, with one module released each week, which included approximately 1 hour of prerecorded lectures. In total, the MEDI 501 course included 23 prerecorded lectures ranging from 15 to 30 minutes on average for 7 hours total lecture content. The course also included two prerecorded interviews with individual scientists from the research team. The prerecorded lectures and interviews were first recorded via Zoom, then integrated with Panopto software for display to the students.

Required assignments for all students seeking a grade and credit for completion consisted of weekly readings, written reflections on the course lectures, and weekly quizzes. Additional recommended readings were also included. All students started out as taking the course for a grade and a small number (i.e., 1–2/session) switched to auditing the course due to competing demands for their time. Students auditing the course still had access to all materials and the ability to fully participate in the course. These students are included in the number of students enrolled and also received the pre- and post-course survey.

Opportunities for interaction included discussion boards in which students responded to one another’s reflections and two live question and answer sessions with faculty where students submitted their questions in advance. These sessions were recorded for later viewing by students who could not participate live. The majority asynchronous design was deliberate to allow for full student participation regardless of work schedule and/or time zone.

MEDI 501 was organized around the overarching learning objectives in Table 1, with lectures and other course materials all reflecting and grounded in these objectives. The full syllabus is available publicly and included in Supplemental Materials, along with additional course materials (i.e., glossary and reading list) [25]. Each week, faculty taught key aspects of the case study, including the scientific and collaborative aspects of conducting the work. They covered critical operational aspects of the project such as the overall project strategy, how goals were established, developing effective team functioning, processes for making key decisions (e.g., stopping or continuing each phase of research and changing directions), and organizational features at NCATS that enabled the project to progress through preclinical research to initiation of a phase 1 clinical trial (Table 2).

**Table 2.** Key topics covered by week in the 2020 MEDI 501 course

Week	Topics	Generalizable TS principles
Week 1	• Introduction to translational science • Overview of the case study from drug discovery to development • Description of how the metarrestin project was initiated	• Address unmet patient needs • Take evidence-informed risks • Pursue major research breakthroughs rather than incremental advances • Use boundary-crossing partnerships
Week 2	• Organizational approaches to enable and optimize effective team-based translational research, innovation, and risk-taking • Creativity and innovation, and evidence-informed risk-taking: phenotypic-based drug discovery approaches and high-throughput screening • Optimizing clinical predictability to accelerate research: in vitro assays for drug discovery and development	• Pursue creativity and innovation • Take evidence-informed risks • Produce cross-cutting solutions • Leverage cross-disciplinary team science • Enhance the efficiency and speed of translational research
Week 3	• Cross-disciplinary collaboration with medicinal chemistry to advance preclinical translation research • Optimize medicinal chemistry contributions to advance from drug discovery to drug development	• Leverage cross-disciplinary team science • Enhance the efficiency and speed of translational research
Week 4	• Approaches for successful cross-agency partnerships (government, academia, and industry), including protecting intellectual property and leveraging legal agreements • Strategies for successful cross-disciplinary team-based science for individuals, teams, and organizations	• Use boundary-crossing partnerships • Leverage cross-disciplinary team science
Week 5	• Aligning animal models with clinical needs to enhance research quality and efficiency • Cross-disciplinary collaboration with pharmacology and toxicology to transition from drug discovery to studies that enable filing for an Investigational New Drug (IND) • Addressing unmet patient needs: focus on metastatic pancreatic cancer	• Address unmet research and patient needs • Leverage cross-disciplinary team science • Enhance the efficiency and speed of translational research
Week 6	• Innovation in preclinical translational research: moving from phenotypically driven studies to target identification	• Pursue creativity and innovation • Enhance the efficiency and speed of translational research
Week 7	• Regulated clinical trials – goals, design, and implementation • Advancing to first in human clinical trials • Wrap up: case study overview from phenotypic observation to drug development and testing, conclusions, and additional resources	• Produce cross-cutting solutions • Review all principles demonstrated in this case

Faculty presented the science in approximately chronological order following the milestones of the project. There was an intentional focus on the interconnectedness of their respective research areas and the handoffs needed to transition to the next phase of the project. The faculty also demonstrated the connections of this work to larger challenges in translational science and their solutions for these challenges, which were highly generalizable to other preclinical research projects. Lectures by experts on central issues in preclinical translational science were interspersed with lectures that focused on the research project, to highlight these topics as they emerged in the story, for example, perspectives on first-in-class drug discovery and development, team science, and clinical trials design. Through these supplementary lectures, as well as interviews with scientists, and live question and answer sessions, the scientific and operational strategies used in this specific case were linked to generalizable translational science principles (Table 2) and approaches to developing solutions to common challenges across a wide variety of research areas [1].

## Course Evaluation Findings: Student Characteristics and Course Satisfaction

Both pre- and post-course surveys were distributed to students to collect information on student characteristics including background relevant to the course, learning goals, degree of participation in the course, effectiveness of the course to teach translational science concepts, and impact of the course on translational science related attitudes, professional activities, and career goals. In total, 100 students responded to either the pre- or post-course survey. This included 95 students who completed the pre-course survey, of whom 66 also completed the post-course survey, and 5 who completed only the post-course survey. This evaluation research was approved by the NIH Institutional Review Board (project number P205038).

Course participants reported a wide range of training and career stages and educational backgrounds (Table 3). More than half of baseline survey respondents had a doctoral degree (60%), and about a third were more than 5 years beyond their highest degree (38%). Survey respondents reported over 30 different degree disciplines, including medicine, biology, chemistry, pharmacology, bioengineering, and public health. Participants worked in a range of sectors, with about two-thirds in academia (67%) and the remainder in other settings, including government, industry, and nonprofit/non-governmental organization (NGO) settings. Regarding course participants’ training and experience relevant to the scientific content covered in the case study, just over half of survey respondents had a background in cancer biology (59%), and about half had a background in drug discovery and development (48%). Finally, most were new to translational research, with about two-thirds (65%) reporting less than 2 years of experience. Additionally, two-thirds (68%) reported that their work at the time of the course contributed to translational research efforts.

**Table 3.** Student characteristics and satisfaction with the course

Baseline student characteristics (n = 95)	No. (%)
*How the student heard about the course*	
Referred by supervisor, principal investigator, mentor, colleague, etc.	28 (29)
Institution/department/program announcement or email	26 (27)
National Institutes of Health (NIH) website, email, or listserv	16 (17)
Foundation for Advanced Education in the Sciences (FAES) website, catalog, or email	12 (13)
Unspecified email/website	9 (10)
Other website (Google, LinkedIn, Twitter)	4 (4)
*Work sector*	
Academia	64 (67)
Government	23 (24)
Industry/business/private sector	3 (3)
Nonprofit/non-governmental organization (NGO)	4 (4)
Other	1 (1)
*Highest degree*	
PhD	48 (51)
Bachelors	24 (25)
Masters	13 (14)
MD	6 (6)
MD/PhD	3 (3)
Other	1 (1)
*Disciplinary training, by highest degree*	
Biology	29 (30)
Medicine	12 (13)
Chemistry	9 (9)
Biochemistry	8 (8)
Other	37 (39)
*Years since highest degree*	
0–5 years	58 (62)
6–10 years	19 (20)
11–15 years	9 (10)
More than 15 years	8 (8)
*Current student*	
Yes	26 (28)
*Translational research (TR) experience*	
Less than 1 year	38 (40)
1–2 years	24 (25)
3–5 years	19 (20)
6–10 years	13 (14)
More than 10 years	1 (1)
*Current work contributes to TR*	
Yes	65 (68)
*Background in cancer biology*	
I have been involved in conducting cancer biology research	32 (33)
I have been involved in conducting other cancer research	31 (32)
I have academic training in cancer biology	29 (30)
I have been involved in providing cancer patient care	10 (10)
None of the above	39 (41)
*Background in drug discovery and development (D&D)*	
I have been involved in conducting drug D&D research	35 (36)
I have academic training in drug D&D	23 (24)
I have been involved in business, administrative, or legal work around drug D&D	10 (10)
None of the above	50 (52)
**End point course satisfaction questions (n = 71)**	**No. (%)**
*Online format effectiveness*	
Somewhat effective	12 (17)
Moderately effective	18 (25)
Very effective	41 (58)
*Case study approach effectiveness*	
Somewhat effective	9 (13)
Moderately effective	12 (17)
Very effective	50 (70)
*Degree to which course achieved its aim to provide a unique window into translational science*	
Slightly	1 (1)
Moderately	22 (31)
Completely	48 (68)
*Overall value of the course to the respondent*	
Slightly valuable	5 (7)
Moderately value	23 (32)
Extremely valuable	43 (61)

At the end of the course, most survey respondents rated the online format (83%) and case study approach (87%) either moderately or very effective (Table 3). In addition, nearly all respondents indicated the course was moderately or extremely valuable to them (93%) and achieved its goal of providing a unique window into translational science (99%). In text responses, students emphasized the value of both the case study-based teaching approach and resources provided during the course. Students cited the quality of the faculty as one reason they rated the value of the course highly. In particular, students mentioned the value of faculty members’ experiential knowledge, transparency about research obstacles and practical strategies for success, and content expertise. Text responses also highlighted the value of the course to individuals with varied degrees of prior exposure to translational science. Exemplar quotes are provided in Table 4.

**Table 4.** Student feedback on value of the course design

Theme	Exemplar quote
Value of case study-based teaching approach	I loved this course and it was a perfect amount of information for a beginner. I greatly appreciated the lecture format of general information combined with the case study. *The case study really helped put the information into context and solidified my understanding of the foundation*.
Value of case study scientists serving as faculty	The course was an *excellent glimpse into the world of translational science as it covered many of its aspects – from drug discovery and bench science to collaboration agreements. Speakers were very transparent and were not afraid to openly talk about obstacles they faced*.
Value of other experts in translational science serving as faculty	While I thought I had a strong background in translational science, this course always had something new to offer every week. I know I will reference the notes and papers from this course in the future. *We rarely talk about effective team science strategies and I think this course brilliantly introduced it.* For the amount of "work" required – I think it’s manageable for nearly any schedule and is useful for all disciplines and career stages.

## Discussion and Future Directions

Enhancing diversity and expanding the translational science workforce will require development and implementation of a range of education activities designed to reach the broad and growing audience of individuals interested in contributing to the field [16]. These activities also will need to capitalize upon the experiential knowledge in the field to identify and disseminate core principles and effective practices that advance the efficiency and impact of all translational research. Here, we have shared our educational philosophy, course objectives (Table 1), course design guided by translational science principles (Table 2), syllabus, and other teaching materials (Supplemental Materials), as well as evaluation findings and student reflections on the course, with the hope they will be useful to inform development of additional translational science courses and educational resources.

Based on the rapid rate at which MEDI 501 reached its full enrollment capacity in both terms it was offered and the broad range of participants who enrolled, there is strong demand from the scientific community for case study-based courses teaching overarching concepts or principles in translational science. Course evaluation results revealed participants found the course was valuable to them and that it provided a unique window into translational science. In a subsequent publication, the NCATS Education Branch will be describing the course evaluation methods in depth and will also share findings specific to change in students’ knowledge and attitudes relevant to translational science, variation in these outcomes by student background, and influence of the course on students’ planned professional activities and career goals in a subsequent report [26].

Participants also provided helpful feedback to inform course improvements, which are ongoing. A revised version of MEDI501 was offered in summer and fall 2021 and included additions to the course content related to translational science principles, team science, and creativity and innovation. In addition, the course evaluation instrument was revised to reflect refinements in how we conceptualize translational science core concepts. Data from the summer and fall 2021 course evaluations are currently being analyzed and will be shared in a future manuscript.

The success of this initial course led the NCATS Education Branch to pursue development of additional online courses, as well as other educational resources (e.g., publications, videos) that leverage case studies from across the translational spectrum to teach translational science concepts. A second course, “MEDI 502: Translational Science in the COVID-19 Pandemic – Accelerating and Enhancing our Response across Preclinical, Clinical, and Population Health Research” launched in fall 2021 and will be offered multiple times in 2022. A third course focused on rare disease research has been piloted with fellows in the NCATS internal research program and will be further developed for an external audience. Each of these case studies provides the opportunity to engage in discussion about a unique set of overarching issues in translational science, including both operational and scientific challenges reflected at different phases of the translational spectrum, and solutions exemplified in that case [1].

These future courses and related educational resources will continue to leverage the experiential knowledge of NCATS staff and our partners by featuring initiatives led or supported by NCATS. One limitation to this approach is that NCATS does not have dedicated teaching faculty, which is in part why course development including incorporating the evaluation approach has been approximately a year-long process (Supplementary Table 1). Faculty members from the NCATS Education Branch were involved in multiple concurrent education and training-related activities. All other faculty dedicated their time to this course out of interest in being part of a new education opportunity developed in collaboration with their colleagues and sharing their knowledge, with no extra incentive for their participation.

Future directions for NCATS education activities also include exploring other venues for sharing this content outside of the formal course structure to reduce potential barriers to participation (e.g., cost, timing of when course is offered, ability to complete course in a specific timeframe) and allow for broader dissemination of the material. As a non-degree-granting institution, NCATS is also pursuing opportunities for external recognition of course completion via microcredentialing. Currently, students have a transcript from FAES as the record of completion.

Educators, institutions, and professional societies may also be interested in developing educational activities focused on core translational science concepts and principles for the broad scientific community. These activities could leverage the research strengths of one organization, or the collective strength of multiple organizations, to develop case studies or utilize other case study-based resources (e.g., publications, training exercises) to share experiential knowledge in translational science. Case studies focused on successful projects that ultimately produce valuable population health benefits are of increasing interest for demonstrating the impact of research investment [27]. Yet, these case studies and others with more proximal outcomes could also be leveraged for educational purposes. Leveraging case study reports that describe the benefits and impact of translational science advances in combination with innovative approaches to education design and delivery would contribute to advancing translational science education. Further, by sharing course designs, evaluation approaches and methods, and evaluation findings in the literature, we will collectively build the evidence base for effective practices in conveying fundamental translational science knowledge to a broad range of individuals comprising the current and future translational science workforce.

## Supplementary material

For supplementary material accompanying this paper visit https://doi.org/10.1017/cts.2022.374. click here to view supplementary material

## References

[1] Austin CP. Opportunities and challenges in translational science. Clinical and Translational Science 2021; 14(5): 1629–1647. DOI 10.1111/cts.13055. 33982407 PMC8504824

[2] Austin CP , Jonson S , Kurilla MG. Foreword to the JCTS COVID-19 special issue. Journal of Clinical and Translational Science 2021; 5(1): e103. DOI 10.1017/cts.2021.400. 34164155 PMC8190713

[3] Brimacombe KR , Zhao T , Eastman RT , et al. An OpenData portal to share COVID-19 drug repurposing data in real time. bioRxiv 2020. DOI 10.1101/2020.06.04.135046.

[4] Brooks PJ , Ottinger EA , Portero D , et al. The platform vector gene therapies project: increasing the efficiency of adeno-associated virus gene therapy clinical trial startup. Human Gene Therapy 2020; 31(19-20): 1034–1042. DOI 10.1089/hum.2020.259. 32993373 PMC7585601

[5] Coussens NP , Sittampalam GS , Jonson SG , et al. The opioid crisis and the future of addiction and pain therapeutics. Journal of Pharmacology and Experimental Therapeutics 2019; 371(2): 396–408. DOI 10.1124/jpet.119.259408. 31481516 PMC6863454

[6] Haendel MA , Chute CG , Bennett TD , et al. The National COVID Cohort Collaborative (N3C): rationale, design, infrastructure, and deployment. Journal of the American Medical Informatics Association 2021; 1(3): 427–443. DOI 10.1093/jamia/ocaa196. PMC7454687 32805036

[7] Low LA , Mummery C , Berridge BR , Austin CP , Tagle DA. Organs-on-chips: into the next decade. Nature Reviews Drug Discovery 2021; 20(5): 345–361. DOI 10.1038/s41573-020-0079-3. 32913334

[8] Sakamuru S , Zhao J , Xia M , et al. Predictive models to identify small molecule activators and inhibitors of opioid receptors. Journal of Chemical Information and Modeling 2021; 61(6): 2675–2685. DOI 10.1021/acs.jcim.1c00439. 34047186 PMC9447431

[9] Adamo JE , Wilhelm EE , Steele SJ. Advancing a vision for regulatory science training. Clinical and Translational Science 2015; 8(5): 615–618. DOI 10.1111/cts.12298. 26083660 PMC5351091

[10] Begg MD , Crumley G , Fair AM , et al. Approaches to preparing young scholars for careers in interdisciplinary team science. Journal of Investigative Medicine 2014; 62(1): 14–25. DOI 10.2310/JIM.0000000000000021. 24169319 PMC3970261

[11] Naar S , Czajkowski SM , Spring B. Innovative study designs and methods for optimizing and implementing behavioral interventions to improve health. Health Psychology 2018; 37(12): 1081–1091. DOI 10.1037/hea0000657. 30307270

[12] Meyers FJ , Begg MD , Fleming M , Merchant C. Strengthening the career development of clinical translational scientist trainees: a consensus statement of the Clinical Translational Science Award (CTSA) Research Education and Career Development Committees. Clinical and Translational Science 2012; 5(2): 132–137. DOI 10.1111/j.1752-8062.2011.00392.x. 22507118 PMC3771692

[13] The National Center for Advancing Translational Sciences. *Translational Science Principles*. (https://ncats.nih.gov/training-education/translational-science-principles).

[14] National Institutes of Health.*Request for Information (RFI): Enhancing the Clinical and Translational Science Awards (CTSA) Program*. (https://grants.nih.gov/grants/guide/notice-files/NOT-TR-19-027.html).

[15] The National Center for Advancing Translational Sciences.*Clinical and Translational Science Awards (CTSA) Program*. (https://ncats.nih.gov/ctsa).

[16] Boulware LE , Corbie G , Aguilar-Gaxiola S , et al. Combating structural inequities — diversity, equity, and inclusion in clinical and translational research. New England Journal of Medicine 2022; 386(3): 201–203. DOI 10.1056/NEJMp2112233. 35029847

[17] Sancheznieto F , Sorkness CA , Attia J , et al. Clinical and translational science award T32/TL1 training programs: program goals and mentorship practices. Journal of Clinical and Translational Sciences 2021, 1–32. DOI 10.1017/cts.2021.884. PMC8826009 35211339

[18] Smyth SS , Coller BS , Jackson RD , et al. KL2 scholars’ perceptions of factors contributing to sustained translational science career success. Journal of Clinical and Translational Sciences 2021, 1–24. DOI 10.1017/cts.2021.886. PMC9003634 35433037

[19] Sorkness CA , Scholl L , Fair AM , Umans JG. KL2 mentored career development programs at clinical and translational science award hubs: practices and outcomes. Journal of Clinical and Translational Science 2020; 4(1): 43–52. DOI 10.1017/cts.2019.424. 32257410 PMC7103475

[20] Barnes LB , Christensen CR , Hansen AJ. Teaching and the Case Method: Text, Cases, and Readings. 3rd ed. Boston, MA: Havard Business School Press, 1994.

[21] Knowles MS , Holton EF , Swanson RA. The Adult Learner. 7th ed. New York: Elsevier, 2011, p. 406.

[22] Greenberg-Worisek AJ , Campbell KA , Klee EW , et al. Case-based learning in translational biomedical research education: providing realistic and adaptive skills for early-career scientists. Academic Medicine 2019; 94(2): 213–216. DOI 10.1097/acm.0000000000002470. 30256254 PMC6351155

[23] Frankowski KJ , Wang C , Patnaik S , et al. Metarrestin, a perinucleolar compartment inhibitor, effectively suppresses metastasis. Science Translational Medicine 2018; 10(441): 246. DOI 10.1126/scitranslmed.aap8307. PMC6176865 29769289

[24] The Foundation for Advanced Education in the Sciences. (https://education.faes.org/).

[25] The National Center for Advancing Translational Sciences. *Translational Science Training and Education Resources*. (https://ncats.nih.gov/training-education/resources).

[26] Vogel AL , Hussain SF , Faupel-Badger JM. Evaluation of an Online Case Study-Based Course in Translational Science for a Broad Scientific Audience: Impacts on Students’ Knowledge, Attitudes, Planned Scientific Activities, and Career Goals. 2022, in press. 10.1017/cts.2022.415 PMC9305082 35949657

[27] Dodson SE , Kukic I , Scholl L , Pelfrey CM , Trochim WM. A protocol for retrospective translational science case studies of health interventions. Journal of Clinical and Translational Science 2020; 5(1): e22. DOI 10.1017/cts.2020.514. 33948245 PMC8057422

