# Supplementary material for: Teaching principles of translational science to a broad scientific audience using a case study approach: A pilot course from the National Center for Advancing Translational Sciences
Source: J Clin Transl Sci. 2022 Mar 21;6(1):e66. doi: 10.1017/cts.2022.374 (PMC9201875; doi:10.1017/cts.2022.374)
Supplement: Supplementary file 1 [file S2059866122003740sup001.pdf]

Supplementary Table 1 – Timeline of Preclinical Translational Science Online Course Development

Year	Milestones	Individuals Involved and Roles*
2018-2019	• May 2018: Publication of peer review publication (STM) about the metarrestin research, putting experiments and data in the public domain<br>• October 2018-February 2019: Planning process to develop seminar series/discussions for NCATS internal fellows that taught translational science core concepts via the metarrestin case study	NCATS Intramural Training Director (JFB) met with members of the metarrestin team (MF, RU, JJM, SP, PST, XX) and NCATS Office of Strategic Alliances (KB) individually and as a group multiple times to plan case-based seminar series for NCATS internal fellows
2019	• March-May 2019: Launched six-week seminar series for NCATS internal fellows. Each week featured a 1 hour to 90-minute session comprising lectures by 2 or more by members of the metarrestin team with framing and discussion by NCATS Intramural Training Director (JFB)<br>• June – December 2019: Based on positive feedback on and reception of internal pilot, developed online course around the metarrestin case study, to reach a broader audience.	JFB, MF, RU, JM, SP, PST, XX, KB<br><br>JFB, MF, RU, JJM, SP, PST, XX, KB, ALV, CPA, MDH, PS, EN
2020	• January-May 2020:<br>• Developed course evaluation study and received IRB exemption for education research<br>• Added course faculty from outside of the metarrestin team, to provide specialized content on relevant translational science concepts (e.g., Science of Team Science, clinical trials design and ethics)<br>• Recorded all faculty lectures<br>• Developed online user interface in partnership with FAES<br>• Developed student assignments, including written reflections and weekly quizzes<br>• Developed online format including lectures, required and recommended readings, and live Q and A sessions with course faculty<br>• June-July 2020: First session of seven-week online MEDI 501 course offered in partnership with FAES, with pre- and post-course student surveys for course evaluation study (50 students)<br>• September-October 2020: Second session of MEDI 501 course offered in partnership with FAES, with	ALV, JFB<br>ALV, CPA, MDH, PS, EN<br>All co-authors<br>ALV, JFB<br>ALV, JFB<br>ALV, JFB<br>ALV, JFB<br>ALV, JFB

	pre- and post-course student surveys for course evaluation study (65 students)	
2021	• January – May 2021:<br>•Completed data analysis from both of the 2020 online course sessions<br>•In response to student feedback, revised the course by adding more content on team science as well as stimulating creativity and innovation. Also in response to student feedback, enhanced interactive nature of student assignments<br>•Revised evaluation instrument to include expanded content on translational science concepts and received approval for IRB application amendment<br>• June-July 2021: Offered first session of updated version of MEDI 501<br>• September-October 2021: Offered second session of updated MEDI 501	ALV, SFH, JFB<br><br>New faculty<br><br>ALV, JFB, SFH<br><br>NCATS Education Branch
Plans for 2022	• Data analysis for course evaluation for the updated version of MEDI 501, and related peer review publication<br>• Launch of microcredentialing program to provide digital badges for completion of NCATS translational science courses	NCATS Education Branch

STM=Science Translational Medicine; NCATS=National Center for Advancing Translational Sciences; IRB=Institutional Review Board; FAES=Foundation for Advanced Education in the Sciences

*Faculty affiliations:

JFB - J.M. Faupel-Badger is Chief of the Education Branch in the Office of Policy, Communications & Education at the National Center for Advancing Translational Sciences, National Institutes of Health, Bethesda, Maryland.

ALV - A.L. Vogel is a Health Science Administrator in the Education Branch in the Office of Policy, Communications & Education at the National Center for Advancing Translational Sciences, National Institutes of Health, Bethesda, Maryland.

SFH - S.F. Hussain is a Presidential Management Fellow in the Education Branch in the Office of Policy, Communications & Education at the National Center for Advancing Translational Sciences, National Institutes of Health, Bethesda, Maryland.

CPA- C. P. Austin was Director of the National Center for Advancing Translational Sciences, National Institutes of Health, Bethesda, Maryland until April 15, 2021 and is now CEO-partner at Flagship Pioneering, Cambridge, Massachusetts.

MDH- M. D. Hall is Chief of the Early Translation Branch, Division of Preclinical Innovation, National Center for Advancing Translational Sciences, National Institutes of Health, Bethesda, Maryland.

EN- E. Ness is Director of the Office of Education and Compliance, Center for Cancer Research, National Cancer Institute, Bethesda, Maryland.

PS - P. Sanderson is a Project Leader in the Therapeutic Development Branch, Division of Preclinical Innovation, National Center for Advancing Translational Sciences, National Institutes of Health, Bethesda, Maryland.

PST- P. S. Terse is a Toxicology Group Leader in the Therapeutic Development Branch, Division of Preclinical Innovation, National Center for Advancing Translational Sciences, National Institutes of Health, Bethesda, Maryland.

XX- X. Xu is a Senior Scientist and Director of Pharmacokinetics in the Therapeutic Development Branch, Division of Preclinical Innovation, National Center for Advancing Translational Sciences, National Institutes of Health, Bethesda, Maryland.

KB - K. Balakrishnan is a Senior Technology Transfer Manager in the Office of Strategic Alliances at the National Center for Advancing Translational Sciences, National Institutes of Health, Bethesda, Maryland.

SP- S. Patnaik is a Chemistry Group Leader in the Early Translation Branch, Division of Preclinical Innovation, National Center for Advancing Translational Sciences, National Institutes of Health, Bethesda, Maryland.

JJM- J. J. Marugan is a Chemistry Group Leader in the Early Translation Branch, Division of Preclinical Innovation, National Center for Advancing Translational Sciences, National Institutes of Health, Bethesda, Maryland.

UR- U. Rudloff is a Senior Investigator, Center for Cancer Research, National Cancer Institute, Bethesda, Maryland.

MF - M. Ferrer is Director of the 3D Tissue Bioprinting Laboratory in the Early Translation Branch, Division of Preclinical Innovation, National Center for Advancing Translational Sciences, National Institutes of Health, Bethesda, Maryland

	pre- and post-course student surveys for course evaluation study (65 students)	
2021	• January – May 2021:<br>•Completed data analysis from both of the 2020 online course sessions<br>•In response to student feedback, revised the course by adding more content on team science as well as stimulating creativity and innovation. Also in response to student feedback, enhanced interactive nature of student assignments<br>•Revised evaluation instrument to include expanded content on translational science concepts and received approval for IRB application amendment<br>• June-July 2021: Offered first session of updated version of MEDI 501<br>• September-October 2021: Offered second session of updated MEDI 501	ALV, SFH, JFB<br>New faculty<br>ALV, JFB, SFH<br>NCATS Education Branch
Plans for 2022	• Data analysis for course evaluation for the updated version of MEDI 501, and related peer review publication<br>• Launch of microcredentialing program to provide digital badges for completion of NCATS translational science courses	NCATS Education Branch

*Faculty affiliations:

JFB - J.M. Faupel-Badger is Chief of the Education Branch in the Office of Policy, Communications & Education at the National Center for Advancing Translational Sciences, National Institutes of Health, Bethesda, Maryland.

ALV - A.L. Vogel is a Health Science Administrator in the Education Branch in the Office of Policy, Communications & Education at the National Center for Advancing Translational Sciences, National Institutes of Health, Bethesda, Maryland.

SFH - S.F. Hussain is a Presidential Management Fellow in the Education Branch in the Office of Policy, Communications & Education at the National Center for Advancing Translational Sciences, National Institutes of Health, Bethesda, Maryland.

CPA- C. P. Austin was Director of the National Center for Advancing Translational Sciences, National Institutes of Health, Bethesda, Maryland until April 15, 2021 and is now CEO-partner at Flagship Pioneering, Cambridge, Massachusetts.

MDH- M. D. Hall is Chief of the Early Translation Branch, Division of Preclinical Innovation, National Center for Advancing Translational Sciences, National Institutes of Health, Bethesda, Maryland.

EN- E. Ness is Director of the Office of Education and Compliance, Center for Cancer Research, National Cancer Institute, Bethesda, Maryland.

PS - P. Sanderson is a Project Leader in the Therapeutic Development Branch, Division of Preclinical Innovation, National Center for Advancing Translational Sciences, National Institutes of Health, Bethesda, Maryland.

PST- P. S. Terse is a Toxicology Group Leader in the Therapeutic Development Branch, Division of Preclinical Innovation, National Center for Advancing Translational Sciences, National Institutes of Health, Bethesda, Maryland.

XX- X. Xu is a Senior Scientist and Director of Pharmacokinetics in the Therapeutic Development Branch, Division of Preclinical Innovation, National Center for Advancing Translational Sciences, National Institutes of Health, Bethesda, Maryland.

KB - K. Balakrishnan is a Senior Technology Transfer Manager in the Office of Strategic Alliances at the National Center for Advancing Translational Sciences, National Institutes of Health, Bethesda, Maryland.

SP- S. Patnaik is a Chemistry Group Leader in the Early Translation Branch, Division of Preclinical Innovation, National Center for Advancing Translational Sciences, National Institutes of Health, Bethesda, Maryland.

JJM- J. J. Marugan is a Chemistry Group Leader in the Early Translation Branch, Division of Preclinical Innovation, National Center for Advancing Translational Sciences, National Institutes of Health, Bethesda, Maryland.

UR- U. Rudloff is a Senior Investigator, Center for Cancer Research, National Cancer Institute, Bethesda, Maryland.

MF - M. Ferrer is Director of the 3D Tissue Bioprinting Laboratory in the Early Translation Branch, Division of Preclinical Innovation, National Center for Advancing Translational Sciences, National Institutes of Health, Bethesda, Maryland

Additional Supplemental Materials include:

MEDI 501: Principles of Preclinical Translational Science:
A Case Study from Cancer Drug Discovery and Development

1.) Brief syllabus with faculty bios
2.) MEDI 501 reading and resources
3.) MEDI 501 course glossary

These materials may be used for education purposes with permission from the authors. Please cite, "Teaching Principles of Translational Science to a Broad Scientific Audience Using a Case Study Approach: A Pilot Course from the National Center for Advancing Translational Sciences" by Faupel-Badger, JM., Vogel, AL, Hussain, SF., et al. 2022

MEDI 501: Principles of Preclinical Translational Science:
A Case Study from Cancer Drug Discovery and Development

Abbreviated syllabus with topics and speakers

2020 Course (Seven weeks)

Course learning objectives:

- Understand the definitions and goals of translational research and translational science, and how they differ.
- Identify a range of scientific and operational principles that can be applied to enhance preclinical translational research projects.
- Learn about the research process necessary to enable a scientific discovery to lead to an effective compound that can be used in humans.
- Learn about the varied roles of different disciplines, as well as agencies -- including industry, government agencies, and academic faculty and institutions -- in advancing translational research, and how to facilitate effective inter-agency and team-based collaboration.

Week 1: Overview of the Course, Translational Science, and Initiation of this Project

Orientation lecture: Introduction to MEDI 501 Principles of Preclinical Translational Science: A Case Study from Cancer Drug Discovery and Development (Jessica Faupel-Badger)

Lecture 1a: Translational Science: Maximizing the Success of Translational Research (Christopher Austin)

Lecture 1b: Challenges in Development of Selective Anti-Metastasis Therapies in Today's Cancer Treatment Landscape(Udo Rudloff)

Lecture 1c: Pursuing Novelty to Accelerate Innovation in Translational Research (Juan Marugan)

Assigned reading, submit questions for speakers/office hours and mini-quiz

Week 2: Optimizing Efficiency and Effectiveness in Translational Research: Infrastructure, Teams and Partnerships, and Scientific Approaches

Lecture 2a: Organizational Approaches to Accelerate Translational Research: NCATS Early Translation Branch (ETB) Capabilities (Matt Hall)

Lecture 2b: *In vitro* Assays for Drug Discovery and Development: Towards Better Clinical Predictability (Marc Ferrer)

Lecture 2c: Using Phenotypic-Based Drug Discovery Approaches to Discover Anti-Metastatic Drugs (Marc Ferrer)

Assigned reading, submit questions for speakers/office hours and mini-quiz

Week 3: Medicinal Chemistry to Advance Preclinical Translational Research

Lecture 3a: Medicinal Chemistry in the Preclinical Translational Research Team (Sam Patnaik)

Lecture 3b: Medicinal Chemistry Approaches in the Metarrestin Project (Sam Patnaik)

Assigned reading, submit questions for speakers/office hours by 11:59pm EDT Wednesday, and mini-quiz

LIVE office hours. Lecturers will answer questions submitted in advance, and reserve time for a few live questions. More information forthcoming, including information on how to join live or view the recording.

Assignments: Week 3 Self-Reflection Discussion Board Post. More information about this will be forthcoming closer to week 3 and included in the instructions for this post.

Week 4: Partnering for Success: Cross-agency Research Alliances and Interdisciplinary Science Teams

Lecture 4a: Partnering for Success, Part 1: Principles and Management of Intellectual Property (Krishna "Balki" Balakrishnan)

Lecture 4b: Partnering for Success, Part 2: Approaches for Effective Collaboration (Krishna "Balki" Balakrishnan)

Lecture 4c: Strategies for Effective Team Interactions: Evidence Based Practices from the Science of Team Science Field (Amanda Vogel)

Lecture 4d: Planning for Success in Team Science (Amanda Vogel)

Assigned reading, submit questions for speakers/office hours, and mini-quiz

Week 5: Advancing Along the Translational Spectrum: Predictive Models in Drug Development; Pharmacology and Toxicology Testing in the Preclinical Research Project

Lecture 5a: Uses, Strengths, and Limitations of Preclinical Cancer Models, Including Animal Models, for Predicting Future Response in Humans (Udo Rudloff)

Lecture 5b: Pancreatic Cancer Overview and Aligning Animal Models with Clinical Needs for the Metarrestin Project (Udo Rudloff)

Lecture 5c: Transitioning from Discovery Research to IND Enabling Studies (Phil Sanderson)

Lecture 5d: Application of Pharmacokinetics in Preclinical Translational Research (Xin Xu)

Lecture 5e: The Role of Toxicology Data in Filing for an Investigational New Drug (IND) (Pramod Terse)

Assigned reading, submit questions for speakers/office hours, and mini-quiz

Week 6: Target Identification

Lecture 6a: Principles for Target Identification in Phenotypic Drug Discovery Efforts (Juan Marugan)

Lecture 6b: Principles for Target Identification in the Metarrestin Project (Juan Marugan)

Assigned reading and mini-quiz

Assignments: Week 6 Self-Reflection Discussion Board Post. More information about this will be forthcoming closer to week 6 and included in the instructions for this post.

Week 7: Regulated Clinical Trials and Course Wrap-Up

Lecture 7a: Clinical Trials Goals, Design and Implementation, Part 1 (Elizabeth Ness)

Lecture 7b: Clinical Trials Goals, Design and Implementation, Part 2 (Elizabeth Ness)

Lecture 7c: Update on Design and Status of NCI 20-C-0023: First-in-Human Phase I Trial to Investigate the Safety, Tolerability, Pharmacokinetics, Biological and Clinical Activity of Metarrestin (ML-246) in Subjects with Metastatic Solid Tumors **(**Udo Rudloff)

Special feature: Discussion with Dr. Sui Huang about the discovery of perinucleolar compartments (PNC), association of PNC with cancer cells, and cancer research (Sui Huang)

Lecture 7d: Course Conclusion and Additional Resources (Jessica Faupel-Badger)

LIVE office hour. Lecturers will answer questions submitted in advance, and reserve time for a few live questions. More information forthcoming, including information on how to join live or view the recording.

Assigned reading and mini-quiz

MEDI 501: Principles of Preclinical Translational Science:
A Case Study from Cancer Drug Discovery and Development

Readings and Resources

2020 Course

Introduction to NCATS

Video Providing an Overview of NCATS: https://www.youtube.com/watch?v=nISQ_9zm5X0 (4 mins)

Video Providing a Look Inside NCATS Labs: https://www.youtube.com/watch?v=FOp-lX3NY6E (7 mins)

Introduction to Translational Science

Austin CP. (2018). Translating translation. *Nature Reviews Drug Discovery*, 17 (7), 455-6.

Population Burden of Cancer

American Cancer Society (ACS) Report: Cancer Facts and Figures, 2020 - https://www.cancer.org/research/cancer-facts-statistics/all-cancer-facts-figures/cancer-facts-figures-2020.html

Siegel RL, Miller KD, and Jemal A. (2020). Cancer statistics, 2020. *CA: A Cancer Journal for Clinicians,* 70(1): 7-30.

General Cancer Biology, and Historical Perspective on Related Research

Hallmarks of Cancer webinar: http://view6.workcast.net/register?pak=4743580399751470 (1 hour)
NOTE: On February 13, 2012, Cell Press hosted this webinar featuring the authors of the Hallmarks of Cancer articles listed below. This recording of the webinar is available after a quick sign-in. It is more accessible than the articles, and thus a great entry point for this content. In the webinar, the authors discuss both the original article and the update.

Hanahan D and Weinberg RA. (2000). The hallmarks of cancer. *Cell,* 100, 57-70.

Hanahan D and Weinberg RA. (2011). Hallmarks of cancer: the next generation. *Cell,* 144, 646-74.

Note: The two articles above represent a 2000 article and its 2011 update. The differences reflect changes in the science in the intervening decade, particularly the emergence of new areas of research.

Current Cancer Treatment Landscape, as Reflected in Recent Drug Approvals

Kurzrock R, Kantarjian HM, Kesselheim AS, and Sigal EV. (2020). New drug approvals in oncology. *Nature Reviews Clinical Oncology*, 17, 140-146.

Singh H, Blumenthal G, and Pazdur R. (2020). Approvals in 2019: international review and a new agnostic molecular entity. *Nature Reviews Clinical Oncology*, 17, 130-132.

Research Papers on Perinucleolar Compartments (PNCs) – Listed Chronologically

Huang S, Deerinck TJ, Ellisman MH, and Spector DL. (1997). The dynamic organization of the perinucleoloar compartment in the cell nucleus. *Journal of Cell Biology*, 137(5): 965-974.

Kamath RV, Thor AD, Wang C, Edgerton SM, Slusarczyk A, Leary DJ, Wang J, Wiley EL, Jovanovic B, Wu Q, Nayar R, Kovarik P, Shi F, and Huang S. (2005). Perinucleolar compartment prevalence has an independent prognostic value for breast cancer. *Cancer Research*, 65(1): 246-53.

Norton JT, Pollock CB, Wang C, Schink JC, Kim JJ, and Huang S. (2008). Perinucleolar compartment prevalence is a phenotypic pancancer marker of malignancy. *Cancer*, 113(4):861-9.

Norton JT, Titus SA, Dexter D, Austin CP, Zheng W, and Huang S. (2009). Automated High-Content Screening for Compounds That Disassemble the Perinucleolar Compartment. *Journal of Biomolecular Screening*, 14(9):1045-53.

Frankowski KJ, Wang C, Patnaik S, Schoenen FJ, Southall N, Li N, Teper Y, Sun W, Kandela I, Hu D, Dextras C, Knotts Z, Bian Y, Norton J, Titus S, Lewandowska MA, Wen Y, Farley KI, Griner LM, Sultan J, Meng Z, Zhou M, Vilimas T, Powers AS, Kozlov S, Nagashima K, Quadri HS, Fang M, Long C, Khanolkar O, Chen W, Kang J, Huang H, Chow E, Goldberg E, Feldman C, Xi R, Kim HR, Sahagian G, Baserga SJ, Mazar A, Ferrer M, Zheng W, Shilatifard A, Aube J, Rudloff U, Marugan JJ, and Huang S. (2018). Metarrestin, a perinucleolar compartment inhibitor, effectively suppresses metastasis. Science Translational Medicine, 16;10(441).

Press related to the release of the Frankowski et al, 2018 paper

National Cancer Institute. (2018). Experimental Cancer Drug Metarrestin Targets Metastatic Tumors. https://www.cancer.gov/news-events/cancer-currents-blog/2018/metaresstin-metastatic-tumors

National Center for Advancing Translational Sciences. (2018). Metarrestin for the Treatment of Pancreatic Cancer. https://ncats.nih.gov/bridgs/projects/active/pancreatic-cancer

National Institutes of Health. (2018). NIH, Northwestern scientists develop potential new approach to stop cancer metastasis. https://www.nih.gov/news-events/news-releases/nih-northwestern-scientists-develop-potential-new-approach-stop-cancer-metastasis

Northwestern University: Paul, M. (2018). A “dirty bomb” battles cancer metastasis. https://news.northwestern.edu/stories/2018/may/a-dirty-bomb-battles-cancer-metastasis/

University of Kansas Cancer Center. (2018). Researchers identify metastasis-suppressing compound. https://www.kucancercenter.org/news-room/news/2018/06/metastasis-suppressing-compound

Phenotypic Screening – Listed Chronologically

Swinney DC and Anthony J. (2011). How were new medicines discovered? *Nature Reviews Drug Discovery*, 10(7),10(7):507-19.

Eder J, Sedrani R, and Wiesmann C. (2014). The discovery of first-in-class drugs: origins and evolution. *Nature Reviews Drug Discovery*, 13(8): 577-87.

Haasen D, Schopfer U, Antczak C, Guy C, Fuchs F, and Selzer P. (2017). How phenotypic screening influenced drug discovery: Lessons from five years of practice. *Assay and Drug Development Technologies*,15(6): 239-246.

Moffat JG, Vincent F, Lee JA, Eder J, and Prunotto M. (2017). Opportunities and challenges in phenotypic drug discovery: an industry perspective. *Nature Reviews Drug Discovery*, 16(8):531-543.

Team Science

Hall KL Vogel AL and Crowston K. (2019). Comprehensive collaboration plans: practical considerations spanning across individual collaborators to institutional supports. In K.L. Hall, A.L. Vogel, and R.T. Croyle (Eds). Strategies for Team Science Success: Handbook of Evidence-Based Principles for Cross-Disciplinary Science and Practical Lessons Learned from Health Researchers. (pp. 587-611). Springer.

Kozlowski SWJ and Bell BS. (2019). Evidence-based Principles and Strategies for Optimizing Team Functioning and Performance in Science Teams. In K.L. Hall, A.L. Vogel, and R.T. Croyle (Eds). Strategies for Team Science Success: Handbook of Evidence-Based Principles for Cross-Disciplinary Science and Practical Lessons Learned from Health Researchers. (pp. 269-293). Springer.

In Vitro vs. In Vivo Research Findings

Johnson JI, Decker S, Zaharevitz D, Rubinstein LV, Venditti JM, Schepartz S, Kalyandrug S, Christian M, Arbuck S, Hollingshead M, Suasville EA. (2001). Relationships between drug activity in NCI preclinical in vitro and in vivo models and early clinical trials. British Journal of Cancer. 18;84(10):1424-31.

Preclinical Drug Development

Padilha E, Wang A, Singleton M, Hughes E, Li D, Rice K, Konrath K, Patnaik, S, Marugan J, Rudloff U, and Xu, X. (2020). Metabolism and pharmacokinetic characterization of metarrestin in multiple species. Cancer Chemotherapy and Pharmacology. DOI: 10.1007/s00280-020-04042-y

Clinical Trials

International Conference on Harmonisation of Technical Requirements for Registration of Pharmaceuticals for Human Use. (2000). *ICH Harmonised Tripartite Guideline. Choice of Control Group and Related Issues in Clinical Trials (E10).* https://database.ich.org/sites/default/files/E10_Guideline.pdf

International Council for Harmonisation of Technical Requirements for Pharmaceuticals for Human Use. (2016). *Guidelines for Good Clinical Practice E6(R2).* https://database.ich.org/sites/default/files/E6_R2_Addendum.pdf

US Office of the Federal Register. (2018). *Electronic Code of Federal Regulations (ECFR) Part 46 - Protection of Human Subjects.* https://www.ecfr.gov/cgi-bin/retrieveECFR?gp=&SID=83cd09e1c0f5c6937cd9d7513160fc3f&pitd=20180719&n=pt45.1.46&r=PART&ty=HTML

United States Food and Drug Administration Clinical Trials Guidance Documents. https://www.fda.gov/regulatory-information/search-fda-guidance-documents/clinical-trials-guidance-documents

Metarrestin Clinical Trial Webpage on ClinicalTrials.Gov –

Metarrestin (ML-246) in Subjects with Metastatic Solid Tumors. https://clinicaltrials.gov/ct2/show/NCT04222413?cond=metarrestin&draw=2&rank=1

MEDI 501: Principles of Preclinical Translational Science:
A Case Study from Cancer Drug Discovery and Development

Course Glossary

Active compound – A compound or ingredient in a formulation that exerts a direct physiological effect on a plant, animal or another microorganism.

Absorption, distribution, metabolism and excretion (ADME) – A process through which a drug administered to a living body is absorbed and enters the systemic circulation, distributed throughout the body, metabolized in the liver and other organs, excreted into urine and bile, etc., and eliminated from the body.

Adverse event/serious adverse event (AE/SAE) **–** Any adverse, unintended sign or symptom, or illness that occurs in a patient who receives a drug. Adverse events include events for which the causal relationship with the drug is unclear. When an adverse event (1) results in death, (2) is life-threatening, requires inpatient hospitalization or causes prolongation of existing hospitalization, results in persistent or significant disability/incapacity, or (3) may have caused a congenital anomaly/birth defect, the event is classified as a serious adverse event. If there is a causal relationship recognized with an AE, it is designated as an adverse reaction or a side effect.

Assay – A test system, used to quantitate the effects of chemical compounds on cellular, molecular or biochemical processes of interest, in drug discovery, development and toxicity testing. The primary assay is the assay used to identify biologically active chemical entities in a screening mode such as a high-throughput screen. Secondary assays are assays performed to determine whether the compounds identified from the primary screen are biologically relevant or artifacts due to assay readout interference. Secondary assays are frequently assays that confirm the activity of compounds in an orthogonal or more complex format (e.g. in cells).

Benefit-risk assessment – Broadly speaking, benefit-risk assessment in FDA's drug regulatory context is making a judgment as to whether the expected benefits (with their uncertainties) of the drug outweigh the potential risks (with their uncertainties and approaches to manage risks) associated with its expected use. Benefit-risk assessment along a product's life cycle – from development through approval and into the post-market setting – can take several forms, for different purposes. Before an investigational drug can be administered in first-in-human clinical trials, FDA must determine that the product will not pose unreasonable risks to the participants. In the context of the marketing authorization of a drug, FDA's regulatory decision-making is based on a determination that the drug is effective and that its expected benefits outweigh its potential risks to patients and to public health.

Biological target – A macromolecule or a set of macromolecules in a biochemical pathway that is hypothesized to play a role in the disease, condition, or outcome of interest. Ideally the target should be druggable so hits, lead compounds, and drug candidates can be identified and optimized to interact with the target. On-target effects refer to impact of the drug when it binds to the predetermined target, while off-target effects describes the effects that can occur when a drug binds to targets other than those for which the drug was meant to bind.

Biomarkers – Naturally occurring or endogenous factors that are objectively measured and evaluated as indicators of normal processes, pathological processes, or pharmacological responses to treatment. Biomarkers that characterize the condition and change of disease and the degree of cure are used as surrogate markers for evaluating the efficacy of new drugs in clinical studies. When clinical studies are conducted, biomarkers also can be used for patient stratification.

Body surface area conversion factor (BSA-CF) – A factor that converts a dose (mg/kg) in an animal species to the equivalent dose in humans (also known as the human equivalent dose), based on differences in body surface. A BSA-CF is the ratio of the body surface area in the tested species to that of an average human.

Chemical lead compound – A chemical compound that has pharmacological or biological activity that is likely to be therapeutically useful, but may nevertheless have a suboptimal structure-activity relationship that requires further modification or improvement. A lead could be selected from a collection of hits or could be a compound that has been optimized from a hit.

> **Optimization of a chemical lead compound –** The optimization process involves design and synthesis steps to improve activity, selectivity, physical properties, pharmacokinetics and safety parameters. Iterative lead optimization often leads to a selected compound that is further evaluated in toxicology studies to determine its potential as a drug candidate.

Chemical library – A collection of compounds that are used in high throughput screening (HTS). Focused libraries to meet specific purposes are strategically designed and created with selected compounds. For example, the Library of Pharmacologically Active Compounds (LOPAC) contains only compounds already known or used in drugs that have already been commercialized.

Clinical trial – Medical research study involving human participants.

> **Phase 0 Clinical Trial –** Exploratory study involving very limited human exposure to the drug, with no therapeutic or diagnostic goals (for example, screening studies, microdose studies).

> **Phase 1 Clinical Trial –** Study that is usually conducted with healthy volunteers and that emphasizes safety and tolerability. The goal is to find out what the drug's most frequent and serious adverse events are and, often, determining what is the exposure (pharmacokinetics) of the drug and how it is absorbed, metabolized and excreted.

> **Phase 2 Clinical Trial –** Study that gathers preliminary data on effectiveness (whether the drug works in people who have a certain disease or condition) and to identify the dose or doses for the Phase 3 studies. For example, participants receiving the drug may be compared with similar participants receiving a different treatment, usually a placebo or a different drug. Safety continues to be evaluated, and short-term adverse events are studied.

> **Phase 3 Clinical Trial –** Pivotal study that gathers more information about safety and effectiveness by studying the drug in larger and more diverse patient populations. A Phase 3 trial may have more than one dosage and may study the drug in combination with other drugs.

Phase 4 Clinical Trial – Study occurring after FDA has approved a drug for marketing, including post-market requirement and commitment studies that are required of or agreed to by the sponsor. These studies gather additional information about a drug's safety, efficacy, or optimal use.

Compound – A substance formed from two or more elements chemically united in fixed proportions.

Control compound – A compound that is routinely run in the same manner as the test compounds in every run of the assay. This term does not refer to the plate controls used to define the maximum and minimum responses, and they may or may not be a “literature standard” or “reference” compound. The objective of using a control compound in an assay is to check if the assay activity/function is reproducible in every run.

Commercialization – The process of bringing new products or services to market. Commercialization entails product development, regulatory approvals, production, distribution, marketing, sales, customer support, and other key functions critical to achieving the commercial success of the new product or service.

De-risking – Involves efforts to reduce the risks, time delays and costs of advancing basic research breakthroughs into treatments. Preclinical research, which connects basic scientific discoveries with initial testing of therapies in humans, is a particularly failure-prone stage of translation. Innovations in drug discovery and development and management of development programs can substantially reduce the risks, time delays and costs of advancing basic research breakthroughs into treatments.

Drug – (1) A substance recognized by an official pharmacopoeia or formulary; (2) A substance intended for use in the diagnosis, cure, mitigation, treatment, or prevention of disease; (3) A substance (other than food) intended to affect the structure or any function of the body; and (4) A substance intended for use as a component of a medicine but not a device or a component, part or accessory of a device. Biological products are included within this definition and are generally covered by the same laws and regulations, but differences exist regarding their manufacturing processes (chemical process versus biological process.)

Drug candidate – A molecule among several that has been shown to have sufficient selectivity and potency toward a target, and favorable medicine-like properties, justifying further development. The drug candidate will then be subjected to a series of tests and studies. At this stage it is not yet a medicine.

Drug discovery and development – The process by which new candidate medications are discovered. Once researchers identify a promising compound for development, they conduct experiments to gather information that will enable them to develop the compound into a medication. This includes research to establish benefits; mechanisms of action; how it is absorbed, distributed, metabolized, and excreted; best means of administration; dosing regimen; toxicity; and interactions with other drugs.

Druggable target – A biological target known or predicted to be able to bind to small molecules that will regulate the function of the target. In recent years, a candidate target for drug development has been regarded as a druggable target when it is expected to bind with high affinity to an antibody drug, small molecule drug, or nucleic acid drug created by using state-of-the art technology.

First in class – An innovative drug that is highly novel and useful and may bring about a significant change to a conventional therapeutic system. Usually refers to the first product with a specific mechanism of action.

First in human (FIH) – The first study in which a study drug (candidate drug substance) is administered to humans. When there are multiple candidate indications, the disease for which efficacy and safety are first evaluated in clinical trials may be referred to as FIH indication.

Food and Drug Administration (FDA or USFDA) – A United States government agency responsible for protecting and promoting public health through the control and supervision of food safety, tobacco products, dietary supplements, prescription and non-prescription drugs (medications), vaccines, biopharmaceuticals, blood transfusions, medical devices, electromagnetic radiation emitting devices, cosmetics, animal food and feed, and veterinary products.

Freedom to Operate (FTO) – The ability of a company to commercialize a specific technology freely. It is important to obtain such Freedom to Operate when the technology is commercialized. "FTO search" refers to searching prior art or existing patents to identify if the potential exists to infringe intellectual property when a specific technology is studied, used, or commercialized.

Genotypic – Relating to an organism's collection of genes, its genotype. The genotype is expressed when the information encoded in the genes' DNA is used to make protein and RNA molecules. The expression of the genotype contributes to the individual's observable traits, called the phenotype.

Good Clinical Practice (GCP) standards – International quality standards for conducting clinical trials, which governments can then transpose into regulations for clinical trials involving human subjects.

Good Laboratory Practice (GLP) standards – A set of standardized practices applied when conducting non-clinical studies. This standard is also used to guarantee that safety study data (such as animal studies) comes from non-clinical studies conducted in accordance with a properly designed protocol when an application for approval is submitted to the regulatory authority. This standard applies to toxicity studies and safety pharmacological studies, which are included in non-clinical studies on the safety of a drug. Among study data to be submitted to the regulatory authority for approval, are studies called GLP-Tox studies.

Good Manufacturing Practice (GMP) standards – Overall requirements for facilities and equipment of manufacturing sites as well as manufacturing control and quality control for manufacture of drugs, etc. GMP standards often include detailed and precise record keeping with proper documentation of source and batches of chemicals, conditions, equipment, and processes used in the manufacturing process.

High throughput screening (HTS) – Throughput refers to the number of samples that can be tested at a time. HTS means many samples can be analyzed in a short period of time. HTS is a technique to evaluate a large number (hundreds of thousands to millions) of compounds in a short period of time using a mechanically controlled, automated system to identify active compounds or hits. Quantitative HTS (qHTS) refers to HTS performed in multiple concentrations to facilitate improved hit selection.

Hit – Active compound discovered during compound screening. This may include hits obtained through primary screening approaches such as HTS and compounds that have been confirmed to be active in secondary, or orthogonal assays. A hit is usually defined by its percent activity at a specific concentration in an assay relative to control compounds. Hit compounds are then chemically optimized

to improve efficacy (i.e., target selectivity, ADME profile, etc.). From this process, a lead compound is identified.

Human equivalent dose (HED) – A dose in humans anticipated to provide the same degree of effect as that observed in animals at a given dose. The term HED is usually used to refer to the human equivalent dose of the NOAEL.

Human subjects research – Research that uses persons as the subjects of experiments or studies.

Intellectual property (IP) – Inventions, ideas, works of art and other products used in commerce developed through creative activities that are legally protected as patents, copyright and trademarks, enabling people to earn recognition or financial benefit from what they invent or create.

> **Intellectual property (IP) rights –** Legal rights to IP. The first type of IP right is a patent right. The party that holds a patent right can execute it exclusively for 20 years from the application (25 years in the pharmaceutical field at the maximum). An "IP strategy" including when and where to apply for a patent and what to patent, is established to gain a competitive advantage in the market. Patent rights are country-specific.

Invention – There are four general requirements for an invention to be patentable (as a utility patent): (1) it must comprise patent eligible subject matter, (2) it must be new, (3) it must be useful, and (4) it must be non-obvious.

Investigational New Drug (IND) Application – An application submitted to the Food and Drug Administration (FDA) to obtain permission to start a clinical study on a new drug or to conduct a study to evaluate the efficacy of an already marketed drug for a new indication. An IND application and FDA's approval to proceed to clinical trial initiation are required for all drug candidates. The IND application includes information on the quality of substances being studied and their efficacy and safety.

In vitro – Research performed outside of a living organism.

In vivo – Research performed in a whole, living organism.

K – A dimensionless factor that adjusts for differences in the surface area to weight ratio of species because of their different body shapes.

K_m – Factor for converting mg/kg dose to mg/m^2 dose.

Kinase – A type of enzyme (a protein that speeds up chemical reactions in the body) that adds chemicals called phosphates to other molecules, such as sugars or proteins. This may cause other molecules in the cell to become either active or inactive. Kinases are a part of many cell processes. Human cells have many different kinases, and they help control important functions, such as cell signaling, metabolism, division, and survival. Certain kinases are more active in some types of cancer cells and blocking them may help keep the cancer cells from growing.

Lead optimization – see the definition above for "optimization of a chemical lead compound"

Licensing – Generally refers to providing access to an invention, IP, or know-how to others or permitting execution rights. A party who licenses the IP right is called a licensor, while the other party who obtains a license is a licensee.

Lowest observed adverse effect level (LOAEL) – The lowest dose tested in an animal species with adverse effects

Maximum recommended starting dose (MRSD) – The highest dose recommended as the initial dose in a clinical trial. In clinical trials of adult healthy volunteers, the MRSD is predicted to cause no adverse reactions. The units of the dose (e.g. mg/kg or mg/m^2) may vary depending on practices employed in the area being investigated

Maximum tolerated dose (MTD) – In a toxicity study, the highest dose in a single or multi-day study that does not produce unacceptable toxicity.

Medicinal chemistry – A discipline at the intersection of chemistry, biology, drug metabolism and pharmacokinetics, and other specialties, involved with the design, chemical synthesis, and evaluation of bio-active small molecules (drugs).

Metastasis – The spread of cancer cells from the place where they first formed to other parts of the body.

Milestone – A milestone is a specific point along a timeline used to manage the progress of a task. It also refers to a defined period in a project schedule or an intermediate goal point. In the pharmaceutical industry, points such as IND application, the end of a Phase I clinical trial, and the end of a Phase II clinical trial are commonly set as milestones, and many agreements provide that a certain payment (milestone fees) be made when a milestone is achieved.

National Center for Advancing Translational Sciences (NCATS) – One of the 27 Institutes and Centers that comprise the US National Institutes of Health. NCATS was created in late 2011 with the goal of transforming the translational science process so that new treatments and cures for disease can be delivered to patients faster. NCATS strives to develop innovations to reduce, remove or bypass costly and time-consuming bottlenecks in the translational research pipeline in an effort to speed the delivery of new drugs, diagnostics and medical devices to patients.

National Institutes of Health (NIH, US NIH) – The primary agency of the United States Government responsible for biomedical and public health research. NIH's mission is to seek fundamental knowledge about the nature and behavior of living systems and the application of that knowledge to enhance health, lengthen life, and reduce illness and disability. The NIH conducts its own scientific research and provides major biomedical research funding to non-NIH research facilities.

New drug application (NDA) – A package of documents required by the FDA before approval is granted for marketing a new drug in the U.S. When the sponsor of a new drug believes that enough evidence on the drug's safety and effectiveness has been obtained to meet the FDA's requirements for marketing approval, the sponsor submits to the FDA a new drug application (NDA). The application must contain data from specific technical viewpoints for review, including chemistry, pharmacology, medical, biopharmaceutics, and statistics. If the NDA is approved, the product may be marketed in the United States.

No observed adverse effect level (NOAEL) – The highest dose at which no toxic and harmful effects were seen in safety studies such as repeated-dose toxicity studies and reproductive and developmental toxicity studies using multiple dose groups.

No observed effect level (NOEL) – The highest dose at which no biological effects cause statistically significant changes in the control group in safety studies such as repeated-dose toxicity studies and reproductive and developmental toxicity studies using multiple dose groups.

Patent – The grant of a property right to the inventor(s) of an invention, issued by the United States Patent and Trademark Office. A U.S. utility patent is generally granted for 20 years from the date the patent application is filed; however, periodic fees are required to maintain the enforceability of the patent. A design patent is generally granted protection for 14 years measured from the date the design patent is granted. Patent rights are country-specific, and separate patent applications have to be filed in each country's patent office. See below for an International Treaty that somewhat simplifies this process, the Patent Cooperation Treaty (PCT).

> **Provisional Patent –** A provisional patent application gives inventors flexibility to quickly protect an invention. A provisional patent application allows one to file without a formal patent claim, oath or declaration, or any information disclosure (prior art) statement. It also allows the term "Patent Pending" to be applied in connection with the description of the invention. A provisional application for patent is valid for 12 months from the date the provisional application is filed. Therefore, an applicant who files a provisional application must file a corresponding nonprovisional application for patent during the 12-month pendency period of the provisional application in order to benefit from the earlier filing of the provisional application.

Patent Cooperation Treaty (PCT) – An international patent law treaty that provides a unified application system in which filing a single patent application allows the applicant to gain the same benefits as those obtained by filing applications in all PCT member countries at the same time. A patent application filed under the PCT is called an international application, or PCT application, and must be filed within 12 months of the provisional patent application. After the PCT application is filed, the applicant has an additional 18 months to choose individual countries where they want to file individual country specific patent applications. This is known as the National Stage.

Perinucleolar Compartment (PNC) – A subnuclear body characterized by its location to the periphery of the nucleolus. The PNC is associated with malignancy both in vitro and in vivo and its presence positively correlates with metastases.

Pharmacodynamics (PD) – The body's biological response to drugs including the drugs' reactions with and binding to cell constituents, and the biochemical and physiological consequences of these actions. PD is commonly related to drug concentration and pharmacokinetic (PK) parameters.

Pharmacokinetics (PK) – The movement of drugs through the body including the process of the uptake of drugs by the body, the biotransformation they undergo, the distribution of the drugs and their metabolites in the tissues, and the elimination of the drugs and their metabolites from the body over a period of time.

Pharmacology – The science of drugs including their origin, composition, modes of action, therapeutic value, and toxicology. The two main areas of pharmacology are pharmacodynamics and pharmacokinetics.

Pharmacologically active dose (PAD) – The lowest dose tested in an animal species with the intended pharmacologic activity.

Phenotypic – Relating to the observable characteristics of an organism, resulting from the interaction of its genotype with the environment.

Post-marketing surveillance – Surveillance conducted to confirm drug efficacy and safety after marketing has been initiated and to collect information on new effects and side effects that were not obtained in pre-marketing clinical trials.

Proof of Mechanism (POM) – Relates to the earliest stages of drug development, often pre-clinical. These studies are designed to show that a new medicine reaches its target organ(s), interacts with its molecular target, and affects the biology of the target cells as intended.

Reproducibility – The extent to which consistent results are obtained when an experiment is repeated, in the same lab or testing the same conditions across different laboratories.

Royalty – A payment received by a licensor who grants the licensee the right to use the patent.

Safety factor – A number by which the human equivalent dose (HED) is divided to introduce a margin of safety between the HED and the maximum recommended starting dose.

Safety margin – The ratio of the clinical dose (exposure level) in humans to the NOAEL (exposure level) obtained from animal toxicity studies, which is used as a parameter for risk assessment in humans. It is calculated as follows: NOAEL/ clinical dose (exposure level) in humans, or exposure level in NOAEL/exposure level in human clinical dose.

Science of Team Science (SciTS) – An interdisciplinary field of study that aims to develop the evidence base for effective practices in team-based science.

Target Product Profile (TPP) – A summary of expected effects, regimen, dose, dosage forms, formulation, adverse reactions etc., for a developed drug. It provides a criterion to evaluate marketability or make a Go/No-go judgment. In recent years, setting the TPP has been required from the early development stage.

Target validation – The use of a variety of approaches to determine the target that a drug or lead compound acts upon. In drug discovery, initial research may focus on a cellular characteristic (e.g. cell death or cell migration) or observable outcome (e.g. protein aggregation, organelle changes) associated with a normal or pathological process without a specific target being identified. Additional experiments are needed to determine what specific component of this process the drug or lead compound in acting upon that alters the observable outcome.

Team science – Scientific research that is conducted by a team of two or more individuals working interdependently to achieve shared scientific goals.

Technology transfer – The process by which valuable research, skills, knowledge, and/or technology is delivered from government, colleges and universities, or other research institutions into the corporate environment where it is developed into a commercial product or service.

Therapeutic index – A quantitative measurement of the relative safety of a drug. It is the ratio of the drug concentration in the blood resulting in toxicity and drug concentration in the blood resulting in efficacy. The larger the therapeutic index the safer the drug.

Therapeutic window – The range of doses achieving the greatest therapeutic benefit without resulting in unacceptable side-effects or toxicity. This range is limited by side effects and treatment effectiveness.

Toxicology – A scientific discipline focused on the study of the adverse effects of chemical substances on living organisms and the practice of diagnosing and treating exposures to toxins and toxicants. The discipline overlaps with biology, chemistry, pharmacology, and medicine.

Translational research – The process of turning observations in the laboratory, clinic, and community into interventions that improve the health of individuals and the public—from diagnostics and therapeutics to medical procedures and behavioral changes.

Translational science – The emerging field of investigation focused on understanding generalizable scientific and operational principles for translational research.

Multiple sources were consulted to develop this glossary. They included:
Assay Guidance Manual
Translational Together
NCATS Website
NCI Dictionary of Cancer Terms
